# Proteomic analysis of s-acylated proteins in human retinal pigment epithelial cells and the role of palmitoylation of Niemann-Pick type C1 protein in cholesterol transport

**DOI:** 10.3389/fnagi.2022.965943

**Published:** 2022-10-03

**Authors:** Jia Kai Li, Yu Qing Rao, Siew Kwan Koh, Peiquan Zhao, Lei Zhou, Jing Li

**Affiliations:** ^1^Department of Ophthalmology, Xinhua Hospital Affiliated to Shanghai Jiao Tong University School of Medicine, Shanghai, China; ^2^Singapore Eye Research Institute, Singapore, Singapore; ^3^Department of Ophthalmology, Yong Loo Lin School of Medicine, National University of Singapore, Singapore, Singapore; ^4^Ophthalmology and Visual Sciences Academic Clinical Research Program, Duke-NUS Medical School, National University of Singapore, Singapore, Singapore

**Keywords:** retinal pigment epithelial cells, palmitoylation, Niemann-Pick type C1 protein, protein post-translational modification, cholesterol transport, acyl-biotin exchange (ABE)

## Abstract

Palmitoylation is a dynamic process that regulates the activity of the modified proteins. Retinal pigment epithelial (RPE) cells play pivotal roles in the visual cycle and maintaining healthy photoreceptor cells. Dysfunctional RPE cells are often associated with degenerative retinal diseases. The aim of the study was to identify potentially palmitoylated proteins in human RPE cells. By using the detergent-resistant membrane, we found 312 potentially palmitoylated peptides which corresponded to 192 proteins in RPE cells, including 55 new candidate proteins which were not reported before. Gene enrichment analysis highlighted significant enrichment of palmitoylated proteins in cell-matrix adhesion, cell-cell recognition, protein cellular localization, and translation, among others. We further studied the effect of 3 potential palmitoylation sites (Cys 799, 900, and 816) of Niemann-Pick type C1 protein (NPC1) on cholesterol accumulation. We found that mutation of any single Cys alone had no significant effect on intracellular cholesterol accumulation while simultaneous mutation of Cys799 and 800 caused significant cholesterol accumulation in the late endosome. No further cholesterol accumulation was observed by adding another mutation at Cys 816. However, the mutation did not alter the cellular localization of the protein. Conclusion: PRE cells have an abundant number of palmitoylated proteins which are involved in cellular processes critical to visual function. The palmitoylation at Cys799 and 800 was needed for cholesterol export, but not the intracellular localization of NPC1.

## Introduction

Protein S-palmitoylation involves the covalent addition of palmitic acid to the cysteine (Cys) residue of the targeted protein. It was estimated that about 10% of the genome encode proteins with palmitoylation (Sanders et al., 2015). Palmitoylation facilitates the anchoring of the modified protein to the lipid membrane. In this way, it modulates protein localization, trafficking, activity, and stability (Linder and Deschenes, 2007; Fukata and Fukata, 2010; Qu et al., 2021; Jansen and Beaumelle, 2022). S-palmitoylation can occur spontaneously or be catalyzed by DHHC domain-containing palmitoy acyltransferases (DHHC proteins or PATs). The palmitoyl moiety can also be cleaved by protein thioesterase (Salaun et al., 2010). The reversibility of S-palmitoylation is unique in protein post-translational modification. For many proteins, the dynamic palmitoylation and de-palmitoylation processes serve to regulate their activity (Linder and Deschenes, 2007; Salaun et al., 2010; Qu et al., 2021; Jansen and Beaumelle, 2022). *In vitro*, protein palmitoylation can also be inhibited by 2-bromopalmitate (2-BP), a palmitate analogue that inhibits PATs and blocks palmitate incorporation by direct covalent competition with the substrate (Davda et al., 2013).

Retinal pigment epithelial cells (RPE cells) are supportive cells of the retina (Sparrow et al., 2010; Yang et al., 2021). Some of the critical contributions of RPE cells to retinal function include the formation of the blood-retina barrier, glucose transportation from choroidal blood vessel to photoreceptor cells, 11-cis-retinal regeneration in the visual cycle, phagocytosis and metabolism of the membranous discs of photoreceptor cells for the renewal of the outer segment. To fulfill these functions, RPE cells are constantly running at high cellular activity and metabolic levels. Knowing the importance of palmitoylation in modulating protein activities, and the extensive participation of palmitoylated proteins in cellular processes, we thought it would be interesting to explore the protein palmitoylation profile in RPE cells.

Niemann-Pick type C1 protein (NPC1) is an intracellular cholesterol transporter localized in the late endosome/lysosome (Pfeffer, 2019). It is responsible for exporting free cholesterol out of lysosome for cellular needs or storage (Rosenbaum and Maxfield, 2011). Mutations in *NPC1* gene are responsible for up to 95% of the Niemann-Pick type C disease (OMIM 257220), a disorder characterized by massive lysosomal accumulation of cholesterol and glycosphingolipids and often manifests with progressive neurodegenerative conditions (Vance, 2006; Percival et al., 2020; Yañez et al., 2020). Cholesterol metabolism is also critical for RPE cell function due to its daily ingestion of lipid-enriched photoreceptor cell membrane (Claudepierre et al., 2010; Yan et al., 2014; Ramachandra Rao and Fliesler, 2021). Mouse carrying point mutation in *Npc1* developed lipofuscin accumulation in the sub-RPE and sub-retina layers and photoreceptor degeneration at the age of 2 months (Claudepierre et al., 2010). Abnormal lipid deposition of RPE cells is also associated with the development of blinding retinal degenerative diseases such as retinitis pigmentosa and age-related macular degeneration (AMD) (Pikuleva and Curcio, 2014; Veleri et al., 2015; Fleckenstein et al., 2021; Lewandowski et al., 2021; Ramachandra Rao and Fliesler, 2021). Although several studies have identified NPC1 as a palmitoylated protein in human and mouse cells and tissues (for a complete list of studies identified NPC1 as palmitoylted protein, see^1^) (Yang et al., 2010; Wilson et al., 2011; Morrison et al., 2015; Sanders et al., 2015; Serwa et al., 2015). The role of palmitoylation on NPC1 activity remains unknown. In this study, we aimed to explore potentially palmitoylated proteins in human RPE cells and validate the effect of palmitoylation on NPC1 protein.

## Materials and methods

### Materials and cells

All chemicals were purchased from Sigma-Aldrich (Merck KGaA, Darmstadt, Germany), unless otherwise specified. The human retinal pigment epithelial cell line ARPE19 was obtained from the American Type Culture Collection. The NPC1 deficient (NPC1*^mut^*, GM03123) and control (NPC1*^wt^*, GM05659) fibroblasts were purchased from Coriell Institute (Coriell Institute for Medical Research, Camden, NJ, United States) (Maziere et al., 1982). Both cell lines were maintained in Dulbecco’s modified Eagle’s medium with 10% FBS, 2 mM L-glutamine, 100 Units/mL penicillin, 100 ug/mL streptomycin in a standard cell culture incubator. All cell culture reagents were purchased from Invitrogen (Thermo Fisher Scientific, Waltham, MA, United States).

### Isolation of detergent-resistant membrane

The detergent-resistant membrane (DRM) was obtained by detergent extraction as previously described (Wan et al., 2007; Yang et al., 2010). Ten 150-mm culture dishes of ARPE19 cells at 90% confluence were used for each experiment. Cells were rinsed three times with cold PBS and scraped off the dish in 4mL ice-cold buffer containing 50 mM HEPES, 10 mMNaCl, 5 mM MgCl_2_ and 0.1 mM EDTA at pH 7.4. The collected cells were homogenized and centrifuged at 500 *g* for 10 min at 4°C to get rid of debris. The crude lysates were spun again at 200,000 *g* for 30 min at 4°C. The resulting pellet was extracted in an equal volume of buffer containing 20 mM2-MES, 150 mM NaCl, 2% Triton X-100 and a cocktail of mixed proteinase inhibitors (Roche Diagnostics Ltd., Basel, Switzerland). After incubation on ice for 60 min, the mixture was spun at 200,000 *g* again for 30 min at 4°C. The pellet was further rinsed in 20 mM MES without Triton X-100 and collected by centrifugation. The resulting pellet was enriched with DRM and ready for labeling.

### Acyl-biotinyl exchange labeling and biotin-streptavidin enrichment of s-acylated protein

The raft-enriched pellet was dissolved in Tris-NaCl buffer containing 60 mM β-octylglucoside and precipitated by chloroform-methanol. The pellet was then completely re-dissolved in 50 mM Tris-HCl with 4% SDS and 5 mM EDTA at pH 7.4 and further diluted with 3 volumes of the same buffer containing 0.2% Triton X-100 without SDS. Tris (2-carboxyethyl) phosphine hydrochloride (TCEP) was added to the above sample to a final concentration of 10 mM and incubated at room temperature (RT) for 30 min with end-over-end rotation. At the end of the incubation, *N*-Ethylmaleimide (NEM) was added to the final concentration of 50 mM. The reaction was carried out at RT for 2.5 h with end-over-end rotation. The resulting protein was precipitated with chloroform-methanol 5 times to remove excess NEM and re-dissolved in Tris buffer containing 4% SDS. This preparation was then divided into two groups: the hydroxylamine (HA)-treated and the mock-treated groups. The HA-treated group was incubated with 1.33 mM Biotin-HPDP and 1 M HA in a Tris-based buffer containing 0.27% Triton X-100 and proteinase inhibitors for 60 min at RT. The mock-treated sample was incubated in the same buffer without HA. At the end of the reaction, the protein was cleansed with chloroform-methanol precipitation and dissolved in 2% SDS buffer at 37°C for 10 min with constant vortexing. The protected, biotin-labeled protein was pooled down by streptavidin-agarose beads in a buffer which contained 50 mM Tris, 150 mM NaCl, 5 mM EDTA, 0.2% Triton X-100 and 0.1% SDS at pH 7.4. After washing, the beads were spun down and the biotin-HPDP residue was cleaved by 20 mM TCEP.

### Western blot

Protein concentrations of freshly separated cellular fractions, including cytosol, non-DRM and DRM, were measured using Micro BCA protein assay kit (Cat. 23235, Thermo Fisher Scientific, Shanghai, China). Ten micrograms of each fraction was used for electrophoresis on SDS-PAGE, transferred to nitrocellulose membrane (Immobilon Western, WBKLS0500, MilliporeSigma, Shanghai, China), probed with antibodies and visualized using chemiluminescent HRP substrate (1620115, Bio-Rad Laboratories, Hercules, CA, United States). The signals were captured by Bio-Rad Gel Doc XR + system and quantified by densitometry. The following antibodies were used at the indicated dilution: anti-GNAI2 at 1:1000 (Cat. PA5-109778, Thermo Fisher Scientific); anti-CAV1 at 1:1000 (Cat. 3238 Cell Signaling Technology, Shanghai, China) 1:1000; anti-β-Tubulin at 1:1000 (Cat. 2146 Cell Signaling Technology) 1:1000.

### In-gel trypsin digestion

The S-acylation enriched proteins obtained above were subjected to SDS-PAGE and the gel was fixed with 50% methanol/10% acetic acid for 1 h at RT and rinsed with distilled water. The lane of interest was cut into 4 slices and transferred to a clean Eppendorf tube. After dehydration using acetonitrile, the gel was soaked in 50 mM NH_4_HCO_3_ with 10 mM DTT for 45 min at RT. After a brief spin to remove the supernatant, acetonitrile was added again to shrink the gel and 55 mM Iodoacetamide (IAA) was added to protect the free–SH group. The reaction was carried out in dark at RT for 45 min. After the incubation, the supernatant was removed and the gel dehydrated again with acetonitrile. The protein was digested in gel with 20 ng/μL trypsin in 2 mM NH_4_HCO_3_ overnight at 37°C with interval mixing. After digestion, the samples were dried by speedvac and kept at −20°C until further analysis.

### Liquid chromatography-tandem mass spectrometry analysis

The digested sample above was reconstituted in 12 μL of loading buffer which contained 0.1% formic acid and 2% acetonitrile. Two microliters of the sample was injected into liquid chromatography-tandem mass spectrometry (LC-MS/MS) system (Ultimate 3000 nanoLC system, Thermo Fisher Scientific) coupled with AB Sciex 5600 TripleTOF (AB Sciex, Framingham, MA, United States)for the analysis. A 15 cm × 75 μm i.d. column packed with Acclaim PepMap RSLC C18 (Thermo Fisher Scientific) was used for reversed phase (RP) separation. This column was connected to a spray tip (New Objectives, Woburn, MA, United States), which was directly coupled with the nano-spray interface into AB Sciex 5600 TripleTOF mass spectrometer. Samples were loaded onto a trap column (Acclaim Pep Map 100 C18, 2 cm × 75 μm i.d., Dionex, Thermo Fisher Scientific) at a flow rate of 5 μL/min. After a 5 min wash with loading buffer (2/98 v/v of acetonitrile/water with 0.1% formic acid), the system was switched into line with the C18 analytical capillary column. A 2-step linear gradient of mobile phase B (2/98 v/v of water/acetonitrile with 0.1% formic acid) from 7 to 60% over 92-min and 60–95% for 3 min at flow rate of 300 nL/min was utilized for this analysis.

Third generation Nanospray Source was installed and other instrumentation settings were as follows: Ionspray Voltage Floating (ISVF) = 2200 V, curtain gas (CUR) = 30, Ion source gas 1 (GS2) = 12, Interface Heater Temperature (IHT) = 125, Declustering potential (DP) = 100 V, Nebuliser current (NC) = 3 for nitrogen gas. All data was acquired using information-dependent acquisition (IDA) mode with Analyst TF 1.7 software (AB Sciex). For IDA parameters, 0.25 s TOF MS survey scan in the mass range of 350–1250 were followed by product ion scan of 0.075 s in the mass range of 100–1500. Switching criteria were set to ions greater than m/z 350 and smaller than m/z 1250 with charge state of 2–5, maximum number of candidate ions to monitor per cycle was 30 spectra and an abundance threshold of >120 counts. Former target ions were excluded for 12 s. IDA Advanced “rolling collision energy (CE),” was required.

### Proteomic data analysis

The peptide sequences appeared in the HA-treated and mock-treated portions were compared. Any sequences that appeared in both portions were removed from the HA-treated portion as they were most likely false-positive. The sequences of S-acylated peptides (with peptide confidence level >95%) were determined. The data was processed using Protein Pilot software 4.5 (AB Sciex) with database search using uniprot_all_Oct2014 (40516 proteins searched). Protein identification was based on false positive rate (FDR) < 1% with 95% peptide confidence level. The web-based portal Metascape^2^ (Version 3.5, released Dec 18, 2021) was used for the enrichment analysis of the putative palmitoylated proteins (Zhou et al., 2019).

### Construction of wildtype and mutated Niemann-Pick type C1 cDNA and transfection

The wildtype (NM_000271) and mutational NPC1 cDNAs were synthesized and the sequences were verified (Genscript, Nanjing, China). All mutated cDNAs were cloned into pcDNA3.1^+^ and used to transiently transfect the *NPC1* deficient (NPC1*^mut^*, GM03123) and control (NPC1*^wt^*, GM05659) fibroblasts using Lipofectamine 3000 (Invitrogen, Thermo Fisher Scientific). The cells were used 3 days after transfection.

To obtain stable lines of transfection, the cDNA of interest was inserted into a lentiviral vector (pHBLV-CMVIE-ZsGreen-Puro, Hanbio Biotechnology, Shanghai, China). Virus preparations of titer higher than 10^9^ were used to transfect NPC1*^wt^* and NPC1*^mut^* cells. Stable transfected cells were selected using 2 μg/mL puromycin.

### Filipin staining of intracellular cholesterol

Filipin staining was carried out as previously described (Jacobs et al., 1997). Cells were grown on poly-*d*-lysine-coated sterile glass coverslips in a 24-well cell culture dish. Prior to staining, cells were rinsed with PBS three times and fixed with fresh-made 3% paraformaldehyde for 1 h at RT. After washing, the cells were incubated with Filipin reagent (Cayman Chemical, Cat. 10009867) at the concentration of 0.05 mg/mL in PBS for 1 h in dark at RT. The cover slip was then rinsed again in PBS and mounted for viewing under a Leica microscope with an excitation wavelength of 360 nm and a bandwidth of 20 nm.

### Quantitation of intracellular cholesterol

Amplex Red cholesterol assay kit (Invitrogen, Thermo Fisher Scientific) was used to measure intracellular cholesterol. Briefly, cells at about 80% confluency in 6-well plates were used. They were washed 3 times in PBS with 1 mg/mL BSA and 2 times in PBS without BSA. For each well, 2 mL of hexane: isopropanol mixture at the ratio of 3:2 was added and incubated for 30 min at RT. After the incubation, the lipid containing supernatant was transferred to a glass tube and the organic solvent was evaporated using a gentle flow of nitrogen. The lipid was extracted again using chloroform and 1% Triton X-100 and dissolved in 1× reaction buffer. The subsequent procedures were carried out according to the instructions provided by the manufacturer. The cell skeleton left on the plate was dissolved in 2 mL of 0.1 N NaOH and protein concentration was measured. This protein concentration was used to standardize the cholesterol concentration for comparison between different groups.

### Statistical analysis

The Statistical Package for the Social Sciences (SPSS) version 21 was used for data analysis. The average and standard error (SE) of data from repeated experiments were presented. Unpaired Student’s *t*-test was used to determine the statistical significance between the two groups. *p*-value that equals or less than 0.05 was considered statistically significant.

## Results

### The experimental design and the enrichment of detergent-resistant membrane in retinal pigment epithelial cells

**FIGURE 1:**
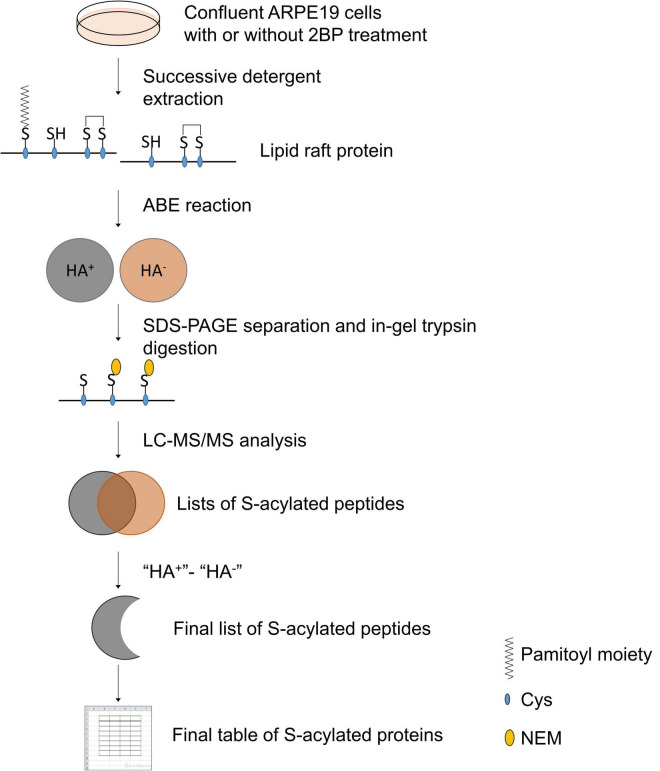
Schematic presentation of experimental design and data analysis.

**FIGURE 2:**
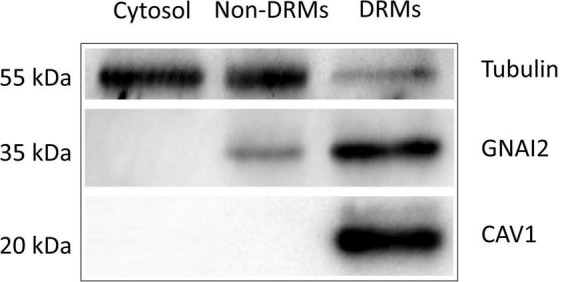
Western blot analysis of Tubulin, GNAI2, and CAV1 proteins in cytoplasmic (Cytosol.), non-detergent-resistant membrane (Non-DRMs), and DRM preparations collected from ARPE19 cell homogenates during successive detergent extraction. Five micrograms of proteins from each preparation were loaded on SDS-PAGE, separated, electro-transferred, and immunoblotted with antibodies against the indicated proteins. The micrographs presented were representative of three independent experiments.

The experimental design and data analysis for palmitoylated protein identification are presented in Figure 1. About 2 × 10^8^ ARPE19 cells were used for the preparation of DRM and the final yield of protein in the DRM fraction was about 4 mg. Figure 2 showed the enrichment of Caveolin-1 (CAV1) and GNAI2 proteins and low content of β-tubulin in the DRM fraction, indicating successful extraction and enrichment of the DRM.

### The identification of potentially palmitoylated proteins

To minimize false-positive results, we set the confidence level of higher than 95% for peptide selection and the false positive rate (FDR) of lower than 1% for protein identification. We also included a mock-treated control at the thioester cleavage step (HA treatment) to identify the non-specific binding of HPDP-biotin in each experiment. Peptides identified in the HA-treated group that were also in the mock-treated group were removed. The results of two independent experiments were compared and the peptides and proteins identified from both experiments were taken as the final potentially palmitoylated targets. In total, we found 312 peptides with palmitoylation site(s), which corresponded to 192 proteins. For a complete list of the proteins and the corresponding peptides, please see Supplementary Table 1. We checked these potential palmitoylation sites against the SwissPalm database^3^ and found 75 peptide sequences that correspond to 55 proteins that were not reported by other groups previously. These peptides were listed in Table 1. Among these proteins are immunoglobulin heavy chain proteins (IGHG2, IGHG3, IGHG4, and IGHM), integrin subunits (ITGB5 and ITGB6). We also identified several palmitoylation target proteins which may have specific functional effects on RPE cells. For example, we found that SLIT3, a transcriptional factor involved in neurogenesis and retina neovascularization was palmitoylated (Ringstedt et al., 2000; Zhou et al., 2017). Another example is oxytocin receptor OXTR. Oxytocin is a nonapeptide that was found in the extracellular matrix of cone photoreceptors (Halbach et al., 2015). Studies have shown that upon binding of oxytocin, OXTR on RPE cells activates a signaling pathway that leads to increased intracellular calcium concentration, and is possibly related to RPE cell trans-activation (Halbach et al., 2015; York et al., 2017; Tsuji et al., 2020).

**TABLE 1:** The list of potentially palmitoylated proteins and peptides identified in this study.

Names	Accession	Gene symbol	Sequence
ATP-binding cassette sub-family A member 2 OS = Homo sapiens GN = ABCA2 PE = 1 SV = 3	Q9BZC7	ABCA2	DAVC*SGQAAARARR
Aldehyde dehydrogenase family 3 member B2 OS = Homo sapiens GN = ALDH3B2 PE = 2 SV = 3	P48448	ALDH3B2	NPC*YVDDNC*DPQTVANR
Aryl hydrocarbon receptor nuclear translocator 2 OS = Homo sapiens GN = ARNT2 PE = 1 SV = 2	Q9HBZ2	ARNT2	EQLC*TSENSMTGRILDLK
Acid-sensing ion channel 3 OS = Homo sapiens GN = ASIC3 PE = 1 SV = 2	Q9UHC3	ASIC3	TC*YLVTQL
A disintegrin and metalloproteinase with thrombospondin motifs 6 OS = Homo sapiens GN = ADAMTS6 PE = 2 SV = 2	Q9UKP5	ADAMTS6	HC*DSPAPSGGGKYC*LGER
DCN1-like protein 3 OS = Homo sapiens GN = DCUN1D3 PE = 1 SV = 1	Q8IWE4	DCUN1D3	AISADSIDGIC*AR
DnaJ homolog subfamily A member 2 OS = Homo sapiens GN = DNAJA2 PE = 1 SV = 1	O60884	DNAJA2	VIEPGC*VR
Desmoglein-2 OS = Homo sapiens GN = DSG2 PE = 1 SV = 2	Q14126	DSG2	SEIQFLISDNQGFSC*PEK
			TLAEVC*LGQK
Dynein heavy chain 12, axonemal OS = Homo sapiens GN = DNAH12 PE = 2 SV = 2	Q6ZR08	DNAH12	WEC*PFDEK
Endothelial protein C receptor OS = Homo sapiens GN = PROCR PE = 1 SV = 1	Q9UNN8	PROCR	EFLEDTC*VQYVQK
Leucine-rich repeat transmembrane protein FLRT2 OS = Homo sapiens GN = FLRT2 PE = 1 SV = 1	O43155	FLRT2	IC*LVPLDAFNYR
Fibronectin type III domain-containing protein 3B OS = Homo sapiens GN = FNDC3B PE = 1 SV = 2	Q53EP0	FNDC3B	LEC*AAAGPQSLK
GRB2-associated and regulator of MAPK protein-like OS = Homo sapiens GN = GAREML PE = 2 SV = 3	Q75VX8	GAREML	GKMPC*LIC*MNHR
GRB2-associated and regulator of MAPK protein OS = Homo sapiens GN = GAREM PE = 1 SV = 2	Q9H706	GAREM	GKMPC*LIC*MNHR
Type I inositol 1,4,5-trisphosphate 5-phosphatase OS = Homo sapiens GN = INPP5A PE = 1 SV = 1	Q14642	INPP5A	SVVETLC*TK
Ig gamma-2 chain C region OS = Homo sapiens GN = IGHG2 PE = 1 SV = 2	P01859	IGHG2	GPSVFPLAPC*SR
			NQVSLTC*LVK
			STSESTAALGC*LVK
Ig gamma-3 chain C region OS = Homo sapiens GN = IGHG3 PE = 1 SV = 2	P01860	IGHG3	GPSVFPLAPC*SR
			NQVSLTC*LVK
			STSGGTAALGC*LVK
Ig gamma-4 chain C region OS = Homo sapiens GN = IGHG4 PE = 1 SV = 1	P01861	IGHG4	GPSVFPLAPC*SR
			NQVSLTC*LVK
			STSESTAALGC*LVK
Ig mu chain C region OS = Homo sapiens GN = IGHM PE = 1 SV = 3	P01871	IGHM	LIC*QATGFSPR
Inhibitor of nuclear factor kappa-B kinase subunit beta OS = Homo sapiens GN = IKBKB PE = 1 SV = 1	O14920	IKBKB	VIYTQLSKTVVC*K
Interleukin-17C OS = Homo sapiens GN = IL17C PE = 1 SV = 1	Q9P0M4	IL17C	LAFAEC*LC*R
Integrin beta-5 OS = Homo sapiens GN = ITGB5 PE = 1 SV = 1	P18084	ITGB5	YQTNPC*IGYK
Integrin beta-6 OS = Homo sapiens GN = ITGB6 PE = 1 SV = 2	P18564	ITGB6	C*DTPANLLAK
Kinesin-like protein KIF26A OS = Homo sapiens GN = KIF26A PE = 2 SV = 3	Q9ULI4	KIF26A	ATAALEQC**VNLC**K
Keratinocyte proline-rich protein OS = Homo sapiens GN = KPRP PE = 1 SV = 1	Q5T749	KPRP	C*PVEIPPIR
Keratin, type I cuticular Ha6 OS = Homo sapiens GN = KRT36 PE = 1 SV = 1	O76013	KRT36	ILDELTLC*K
Target of rapamycin complex subunit LST8 OS = Homo sapiens GN = MLST8 PE = 1 SV = 1	Q9BVC4	MLST8	NIASVGFHEDGRWMYTGGEDC*TAR
Lysozyme C OS = Homo sapiens GN = LYZ PE = 1 SV = 1	P61626	LYZ	GISLANWMC*LAK
			TPGAVNACHLSC*SALLQDNIADAVAC* AK
Transcription factor Maf OS = Homo sapiens GN = MAF PE = 1 SV = 2	O75444	MAF	KEPVETDRIISQC*GR
Methionine aminopeptidase 1 OS = Homo sapiens GN = METAP1 PE = 1 SV = 2	P53582	METAP1	NC*YPSPLNYYNFPK
Malectin OS = Homo sapiens GN = MLEC PE = 1 SV = 1	Q14165	MLEC	VC*ALYIMAGTVDDVPK
Protein MMS22-like OS = Homo sapiens GN = MMS22L PE = 1 SV = 3	Q6ZRQ5	MMS22L	IIDC*LLLPHAVLQQEK
Myelin regulatory factor OS = Homo sapiens GN = MYRF PE = 1 SV = 3	Q9Y2G1	MYRF	SSSVVPDQAC*ISQR
Ig mu heavy chain disease protein OS = Homo sapiens PE = 1 SV = 1	P04220		LIC*QATGFSPR
Myosin-IIIa OS = Homo sapiens GN = MYO3A PE = 2 SV = 2	Q8NEV4	MYO3A	LILIQAC*VR
Nuclear apoptosis-inducing factor 1 OS = Homo sapiens GN = NAIF1 PE = 1 SV = 1	Q69YI7	NAIF1	VNAVATC*RR
Neurogenic locus notch homolog protein 2 OS = Homo sapiens GN = NOTCH2 PE = 1 SV = 3	Q04721	NOTCH2	DTYEC*TC*QVGFTGK
			NC*QTLVNLC*SR
Oxytocin receptor OS = Homo sapiens GN = OXTR PE = 1 SV = 2	P30559	OXTR	VEVAVLC*L
Pyrroline-5-carboxylate reductase 1, mitochondrial OS = Homo sapiens GN = PYCR1 PE = 1 SV = 2	P32322	PYCR1	SLLINAVEASC*IR
Basement membrane-specific heparan sulfate proteoglycan core protein OS = Homo sapiens GN = HSPG2 PE = 1 SV = 4	P98160	HSPG2	LLQVTPADSGEYVC*R
Polymeric immunoglobulin receptor OS = Homo sapiens GN = PIGR PE = 1 SV = 4	P01833	PIGR	QSSGENC*DVVVNTLGK
Rho-related GTP-binding protein RhoH OS = Homo sapiens GN = RHOH PE = 1 SV = 1	Q15669	RHOH	C*VLVGDSAVGK
60S ribosomal protein L10-like OS = Homo sapiens GN = RPL10L PE = 1 SV = 3	Q96L21	RPL10L	LIPDGC*GVK
Ras-related protein R-Ras OS = Homo sapiens GN = RRAS PE = 1 SV = 1	P10301	RRAS	IC*SVDGIPAR
SHC SH2 domain-binding protein 1 OS = Homo sapiens GN = SHCBP1 PE = 1 SV = 3	Q8NEM2	SHCBP1	LAEPYLC*DSQVSTFTMEC* MKELLDLK
Slit homolog 3 protein OS = Homo sapiens GN = SLIT3 PE = 2 SV = 3	O75094	SLIT3	C*SNKGLR
Schlafen family member 11 OS = Homo sapiens GN = SLFN11 PE = 1 SV = 2	Q7Z7L1	SLFN11	QKLVNMGGYTGKVC*VR
Schlafen family member 13 OS = Homo sapiens GN = SLFN13 PE = 2 SV = 1	Q68D06	SLFN13	QKLVNMGGYTGKVC*VR
			VKAFC*C*VVFSEAPK
Synaptonemal complex protein 2-like OS = Homo sapiens GN = SYCP2L PE = 1 SV = 2	Q5T4T6	SYCP2L	RPFNSENAKKAPDC*LIK
Lactotransferrin OS = Homo sapiens GN = LTF PE = 1 SV = 6	P02788	LTF	C*GLVPVLAENYK
			FDEYFSQSC*APGSDPR
			FFSASC*VPGADK
			LADFALLC*LDGK
			NLLFNDNTEC*LAR
			SVQWC*AVSQPEATK
Melanotransferrin OS = Homo sapiens GN = MFI2 PE = 1 SV = 2	P08582	MFI2	C*LAEGAGDVAFVK
			GDSSGEGVC*DKSPLER
			GLLC*DPNRLPPYLR
			GTSADHC*VQLIAAQEADA ITLDGGAIYEAGK
			LSVMGC*DVLK
			WC*VLSTPEIQK
von Willebrand factor D and EGF domain-containing protein OS = Homo sapiens GN = VWDE PE = 2 SV = 4	Q8N2E2	VWDE	C*VGPSTC*SC*PSGWSGKR
WD repeat-containing protein 47 OS = Homo sapiens GN = WDR47 PE = 1 SV = 1	O94967	WDR47	VHC*FEEAC*VMVAEFIPADRK
YjeF N-terminal domain-containing protein 3 OS = Homo sapiens GN = YJEFN3 PE = 1 SV = 1	A6XGL0	YJEFN3	QQLVELC*GHASAVAVTK
Zinc finger protein 646 OS = Homo sapiens GN = ZNF646 PE = 1 SV = 1	O15015	ZNF646	RHC*C*SIC*GKAFR

The potentially palmitoylated Cys residues were marked with *.

**FIGURE 3:**
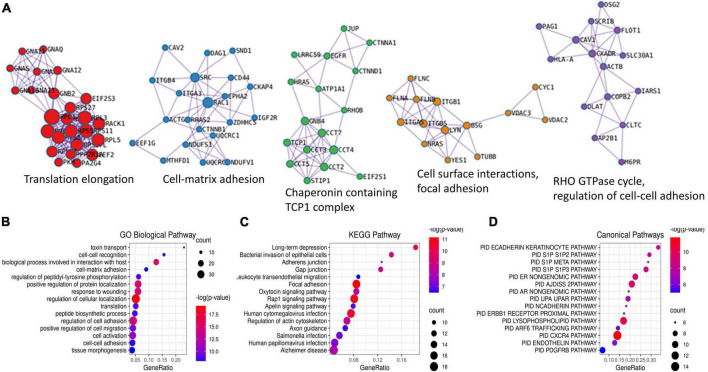
Enrichment analysis of all potentially palmitoylated proteins identified in RPE cells using Metascape. Five MCODE complexes were identified **(A)**. The top 15 most significant Gene Ontology (GO) biological pathways **(B)**, KEGG pathways **(C)**, and Canonical pathways **(D)** were presented by bubble graphs. The color and size of each bubble indicated the value of –log(*p*-value) and enriched gene counts, respectively.

To better understand the significance of palmitoylation on RPE activity, we subjected the above list of 192 proteins to enrichment analysis using Metascape. The analysis identified 5 MCODE complexes, concentrated on protein translation, cell-matrix adhesion, chaperonin containing TCP1 complex, regulation of cell-cell adhesion, cell surface interactions, and focal adhesion (Figure 3A). The over-represented gene ontology terms with the most significant *P*-values were the regulation of cellular localization and biological process involved in interaction with the host. This is consistent with the fact that human S-palmitoylation is highly involved in protein-membrane interaction and pathogen invasion (Ning et al., 2021). Other over-represented GO terms included cell-cell adhesion, cell-matrix adhesion, regulation of cell migration, response to wounding, cell activation, translation, toxin transport, and tissue morphogenesis (Figure 3B). Consistently, the top enriched KEGG pathways included focal adhesion, human cytomegalovirus infection, bacterial invasion of epithelial cells, regulation of actin cytoskeleton, gap junction and adherens junction (Figure 3C). Other enriched KEGG pathways included various synapses, which reflected the role of palmitoylation in neural synaptic plasticity (Fukata and Fukata, 2010; Ji and Skup, 2021). Overall, the enriched processes and pathways are in good agreement with the results obtained using collated S-palmitoylated proteins, which further supported the reliability of our data (Ning et al., 2021). Furthermore, the enriched functions also included several pathways which have unique importance to RPE cells in the retina. For example, a query on the pathway interaction database revealed the CXCR4 pathway as the top enriched pathway among all (Figure 3D). CXCR4 is the predominant chemokine receptor expressed on RPE cells (Crane et al., 2000). In response to its ligand stromal cell-derived factor 1a (SDF-1a), CXCR4 modulates the effects of chronic inflammation and subretinal neovascularization at the RPE site (Crane et al., 2000). CXCR4 expression in RPE cells increased in aged human eyes and in eyes with age-related macular degeneration (Bhutto et al., 2006). Our findings suggested another mechanism that could potentially affect CXCR4 signaling in RPE cells. RAP1 signaling is another KEGG pathway that was over-represented by the palmitoylated proteins in RPE cells. A series of studies from multiple groups showed that RAP1 signaling in RPE cells regulates RPE cell barrier function and plays a critical role in reversing inflammatory cytokine- and VEGF-induced RPE permeability (Wittchen and Hartnett, 2011; Wang et al., 2014, 2016; Li et al., 2018). Our findings again suggested palmitoylation as another mechanism that could potentially affect RAP1 signaling.

2-Bromopalmitate was known to inhibit protein palmitoylation by direct substrate competition and by the inhibition of PATs. To further validate and discern the potential palmitoylation sites detected above, we treated ARPE19 cells with 50 mM 2BP for 24 h in tissue culture media and harvested the cells for the identification of potentially palmitoylated proteins using the same approach as above. Theoretically, proteins with a turn-over time longer than 24 h and stable palmitoylation would be identified in 2-BP-treated cells. Consistent with its inhibitory functions, only 116 peptides and 65 proteins were identified from 2BP-treated cells (Supplementary Table 2). Among these, 49 peptides of 28 proteins were also found in cells without 2-BP treatment, suggesting that these cysteines were likely stably palmitoylated (Table 2). These proteins included caveolin-1, various guanine nucleotide-binding protein G subunits, calpain-5, and ion-channel proteins. Gene ontology analysis revealed that they are significantly enriched in adenylate cyclase-modulating G protein-coupled receptor signaling pathway, G protein-coupled acetylcholine receptor signaling pathway, plasma membrane organization, and membrane fusion (Table 3). Collectively, the results depicted the importance of protein palmitoylation for RPE cell function.

**TABLE 2:** Proteins and peptides that are identified in both control and 2-BP-treated ARPE-19 cells.

Accession	Protein code	Full name	Peptide with potential palmitoylation site
P50895	BCAM_HUMAN	Basal cell adhesion molecule OS = Homo sapiens GN = BCAM PE = 1 SV = 2	EGDEVTLIC*SAR
			EGDTVQLLC*R
O15484	CAN5_HUMAN	Calpain-5 OS = Homo sapiens GN = CAPN5 PE = 1 SV = 2	KPEDEVLIC*IQQRPK
			LAC*GLVK
Q03135	CAV1_HUMAN	Caveolin-1 OS = Homo sapiens GN = CAV1 PE = 1 SV = 4	SFLIEIQC*ISR
			VYSIYVHTVC*DPLFEAVGK
P51636	CAV2_HUMAN	Caveolin-2 OS = Homo sapiens GN = CAV2 PE = 1 SV = 2	SVTDVIIAPLC*TSVGR
Q07065	CKAP4_HUMAN	Cytoskeleton-associated protein 4 OS = Homo sapiens GN = CKAP4 PE = 1 SV = 2	SSSSSSASAAAAAAAASSSASC*SR
P78310	CXAR_HUMAN	Coxsackievirus and adenovirus receptor OS = Homo sapiens GN = CXADR PE = 1 SV = 1	GETAYLPC*K
Q8IWE4	DCNL3_HUMAN	DCN1-like protein 3 OS = Homo sapiens GN = DCUN1D3 PE = 1 SV = 1	AISADSIDGIC*AR
O60884	DNJA2_HUMAN	DnaJ homolog subfamily A member 2 OS = Homo sapiens GN = DNAJA2 PE = 1 SV = 1	VIEPGC*VR
Q14126	DSG2_HUMAN	Desmoglein-2 OS = Homo sapiens GN = DSG2 PE = 1 SV = 2	SEIQFLISDNQGFSC*PEK
			TLAEVC*LGQK
Q9UNN8	EPCR_HUMAN	Endothelial protein C receptor OS = Homo sapiens GN = PROCR PE = 1 SV = 1	EFLEDTC*VQYVQK
P29992	GNA11_HUMAN	Guanine nucleotide-binding protein subunit alpha-11 OS = Homo sapiens GN = GNA11 PE = 1 SV = 2	AC*C*LSDEVK
			IIYSHFTC*ATDTENIR
			TLWEDPGIQEC*YDR
Q14344	GNA13_HUMAN	Guanine nucleotide-binding protein subunit alpha-13 OS = Homo sapiens GN = GNA13 PE = 1 SV = 2	FLVEC*FR
P63096	GNAI1_HUMAN	Guanine nucleotide-binding protein G(i) subunit alpha-1 OS = Homo sapiens GN = GNAI1 PE = 1 SV = 2	IIHEAGYSEEEC*KQYK
			IQC*QFEDLNK
P04899	GNAI2_HUMAN	Guanine nucleotide-binding protein G(i) subunit alpha-2 OS = Homo sapiens GN = GNAI2 PE = 1 SV = 3	ITHSPLTIC*FPEYTGANK
			ITHSPLTIC*FPEYTGANKYDEAASYIQSK
			LWADHGVQAC*F
			QLFALSC*TAEEQGVLPDDLSGVIR
			RLWADHGVQAC*F
			SC*TAEEQGVLPDDLSGVIR
			TIC*FPEYTGANK
P08754	GNAI3_HUMAN	Guanine nucleotide-binding protein G(k) subunit alpha OS = Homo sapiens GN = GNAI3 PE = 1 SV = 3	DGGVQAC*FSR
			IQC*QFEDLNR
P50148	GNAQ_HUMAN	Guanine nucleotide-binding protein G(q) subunit alpha OS = Homo sapiens GN = GNAQ PE = 1 SV = 4	AC*C*LSEEAK
			IIYSHFTC*ATDTENIR
			SLWNDPGIQEC*YDR
P63092	GNAS2_HUMAN	Guanine nucleotide-binding protein G(s) subunit alpha isoforms short OS = Homo sapiens GN = GNAS PE = 1 SV = 1	HYC*YPHFTC*AVDTENIR
			SNEYQLIDC*AQY
			SNEYQLIDC*AQYFLDK
			SNEYQLIDC*AQYFLDKIDVIK
Q14642	I5P1_HUMAN	Type I inositol 1,4,5-trisphosphate 5-phosphatase OS = Homo sapiens GN = INPP5A PE = 1 SV = 1	SVVETLC*TK
P26006	ITA3_HUMAN	Integrin alpha-3 OS = Homo sapiens GN = ITGA3 PE = 1 SV = 5	AKSETVLTC*ATGR
Q14165	MLEC_HUMAN	Malectin OS = Homo sapiens GN = MLEC PE = 1 SV = 1	VC*ALYIMAGTVDDVPK
Q9NZM1	MYOF_HUMAN	Myoferlin OS = Homo sapiens GN = MYOF PE = 1 SV = 1	ASLLSAPPC*R
			ELPDSVPQEC*TVR
Q9NRY6	PLS3_HUMAN	Phospholipid scramblase 3 OS = Homo sapiens GN = PLSCR3 PE = 1 SV = 2	VETFLGWETC*NR
Q8NFJ5	RAI3_HUMAN	Retinoic acid-induced protein 3 OS = Homo sapiens GN = GPRC5A PE = 1 SV = 2	ATTVPDGC*R
P10301	RRAS_HUMAN	Ras-related protein R-Ras OS = Homo sapiens GN = RRAS PE = 1 SV = 1	IC*SVDGIPAR
P62070	RRAS2_HUMAN	Ras-related protein R-Ras2 OS = Homo sapiens GN = RRAS2 PE = 1 SV = 1	KFQEQEC*PPSPEPTRK
O00161	SNP23_HUMAN	Synaptosomal-associated protein 23 OS = Homo sapiens GN = SNAP23 PE = 1 SV = 1	TTWGDGGENSPC*NVVSK
P08582	TRFM_HUMAN	Melanotransferrin OS = Homo sapiens GN = MFI2 PE = 1 SV = 2	GLLC*DPNRLPPYLR
Q9Y277	VDAC3_HUMAN	Voltage-dependent anion-selective channel protein 3 OS = Homo sapiens GN = VDAC3 PE = 1 SV = 1	SC*SGVEFSTSGHAYTDTGK
			VC*NYGLTFTQK

The potential palmitoylated cysteine residues are marked with *.

**TABLE 3:** Top GO processes that were overrepresented by proteins with potential 2-BP in-sensitive palmitoylation sites.

Term	Description	LogP	Log(*q*-value)
GO:0007188	adenylate cyclase-modulating G protein-coupled receptor signaling pathway	−8.8663642	−5.842
GO:0007213	G protein-coupled acetylcholine receptor signaling pathway	−6.164388229	−3.581
GO:0050878	regulation of body fluid levels	−6.004335142	−3.435
GO:1901699	cellular response to nitrogen compound	−5.825621334	−3.298
GO:0095500	acetylcholine receptor signaling pathway	−5.545152449	−3.043
GO:0007212	dopamine receptor signaling pathway	−5.501218442	−3.035
GO:0098901	regulation of cardiac muscle cell action potential	−5.501218442	−3.035
GO:1905145	cellular response to acetylcholine	−5.458736113	−3.004
GO:1905144	response to acetylcholine	−5.339117067	−2.901
GO:0098926	postsynaptic signal transduction	−5.195206554	−2.767
GO:0007189	adenylate cyclase-activating G protein-coupled receptor signaling pathway	−5.068209908	−2.678
GO:0007009	plasma membrane organization	−5.032073786	−2.649
GO:0042060	wound healing	−4.917704633	−2.553
GO:0061025	membrane fusion	−4.883994703	−2.528
GO:0042391	regulation of membrane potential	−4.386805856	−2.097

### Palmitoylation defect in Niemann-Pick type C1 is associated with increased intracellular cholesterol accumulation in the lysosome

**FIGURE 4:**
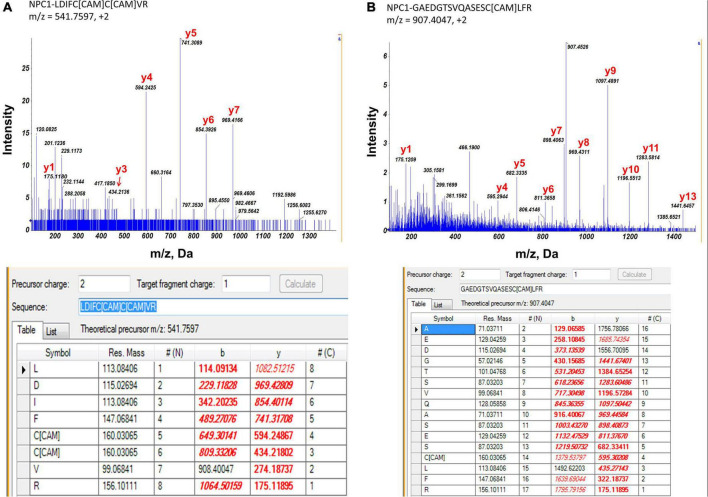
MS/MS spectrum of two peptide fragments originated from NPC1 protein. *Carboxyamidomethylcysteine. **(A)** Peptide fragment LDIFC*C*VR (m/z = 541.8, charge = 2+). It showed evidence of palmitoylation at Cys799 and Cys800 of NPC1 protein. **(B)** Peptide fragment GAEDTSVQASESC*LFR (m/z = 907.4, charge = 2+). It showed evidence of palmitoylation at Cys816 of NPC1 protein. Experimental data matched with theoretical values were highlighted in red.

As photoreceptor cells shed about 7% of their outer segment every day, it makes RPE cells the most active phagocytes in the body (Kwon and Freeman, 2020). Digesting and metabolizing a heavy load of the lipid-rich membranous structure requires tight coordination of multiple processes in the RPE cell. NPC1 at the lysosomal membrane serves as a cholesterol transporter and plays an important role in RPE cell cholesterol clearance. In this study, we identified 3 potential palmitoylation sites in two peptides: LDIFC*C*VR (m/z = 541.8, charge = 2+, Cys^799^ and Cys^800^) and GAEDTSVQASESC*LFR (m/z = 907.4, charge = 2 + , Cys^816^) (Figure 4). Palmitoylation at Cys^799^ and Cys^800^ were also identified by several other studies and predicted by CSS-Palm (CSS-Palm, version 4.0) (Yang et al., 2010; Wilson et al., 2011; Morrison et al., 2015; Sanders et al., 2015; Serwa et al., 2015). However, there were no reports on the role of palmitoylation on NPC1 protein activity.

To test the potential function of Cys palmitoylation, we mutated Cys residue at 799, 800, and 816 to Ala individually and in combination, and constructed 5 expression vectors that express the following mutated NPC1 cDNA: NPC1 a.799 C > A, NPC1 a.800 C > A, NPC1 a.816 C > A, NPC1 a.799/800 C > A, and NPC1 a.799/800/816 C > A.

**FIGURE 5:**
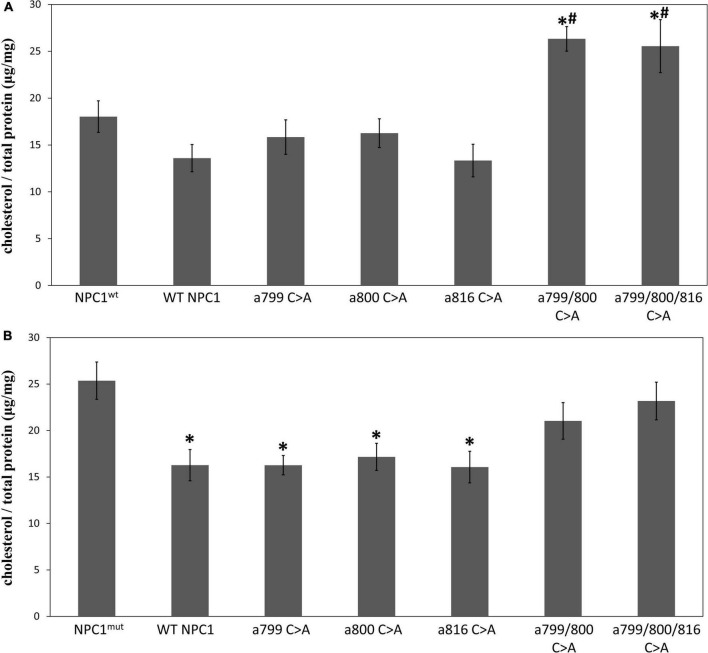
The effect of mutated NPC1 proteins on cholesterol accumulation. Intracellular cholesterol levels were measured as described and expressed as μg per mg total cellular protein. **(A)** NPC1^wt^ cells were transfected with pcDNA3.1 containing wildtype (WT NPC1) or mutated NPC1 cDNA at the indicated positions. *Indicates a significant difference when compared to NPC1^wt^ (*p* < 0.05 by unpaired *t*-test, the same for the rest of the comparisons). ^#^Indicates a significant difference when compared to WT NPC1. **(B)** NPC1^mut^ cells were transfected with pcDNA3.1 containing wildtype (WT NPC1) or mutated NPC1 cDNA at the indicated positions. *Indicates a significant difference when compared to NPC1^mut^. The transfection experiments were repeated three times with triplicated wells each time and the cholesterol levels were measured in duplicates. The average value for each condition was calculated and presented. Error bars stand for standard error means.

We first introduced these mutated cDNAs into fibroblast cells with intrinsic NPC1 gene deficiency (GM03123, NPC1*^mut^*) and the matching controls (GM05659, NPC1*^wt^*), and measured the intracellular cholesterol contents (Figure 5). As expected, the NPC1*^mut^* fibroblasts contained higher intracellular cholesterol levels than NPC1*^wt^* cells. When NPC1*^wt^* was transfected with wildtype NPC1 protein (Figure 5A), we observed a small decrease of intracellular cholesterol. The introduction of a single Cys mutation had no significant effect on the cholesterol level in NPC1*^wt^* cells. However, the introduction of double and triple Cys mutated NPC1 into NPC1*^wt^* cells caused a small yet significant increase of cholesterol, suggesting that the expression of mutated protein interfered with endogenous wildtype protein and hindered cholesterol transport. On the other hand, the expression of wildtype NPC1 cDNA in NPC1*^mut^* significantly reduced intracellular cholesterol levels (Figure 5B). The introduction of single Cys mutations to NPC1*^mut^* fibroblasts also lowered cholesterol levels to concentrations similar to that of the NPC1*^wt^* cells, suggesting that these proteins had similar cholesterol transport activity as the wildtype protein. However, when NPC1*^mut^* was transfected with NPC1 a.799/800 C > A, we observed no reduction in cholesterol, suggesting that the Cys 799/800 double mutation rendered NPC1 inactive. The introduction of triple mutation NPC1 a.799/800/816 C > A to NPC1*^mut^* cells did not cause further reduction of cholesterol.

**FIGURE 6:**
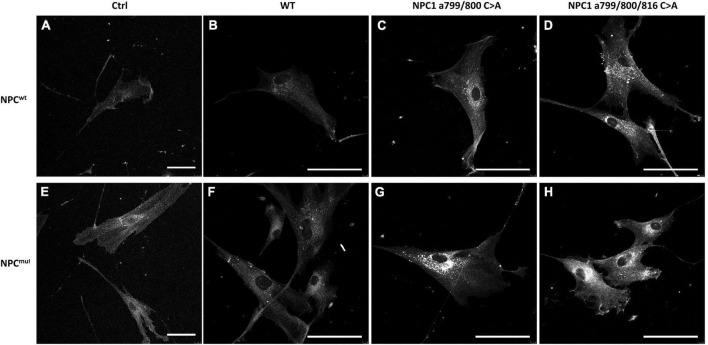
Filipin staining of intracellular cholesterol in NPC1^wt^ (Top panels **A–D**) and NPC1^mut^ fibroblasts (Bottom panels **E–H**) transfected with wildtype (WT NPC1) and mutated NPC1 proteins as indicated. The introduction of wildtype NPC1 protein alleviated cholesterol accumulation in NPC1^mut^ cells (Panel **F**). The introduction of double and triple-mutated NPC1 caused significant cholesterol accumulation in both cells (Panels **C,D,G,H**). The scale bar denotes 50 μm.

To validate that the changes of intracellular cholesterol levels were in fact due to cholesterol accumulation in the lysosome, we performed Filipin staining on stable lines of NPC1 a.799/800 C > A and NPC1 a.799/800/816 C > A transfected NPC1*^wt^* and NPC1*^mut^* cells. The results were shown in Figure 6. Intracellular cholesterol accumulation was visible in NPC1*^mut^* but not in NPC1*^wt^* cells (Figures 6A,E). The introduction of wildtype NPC1 cDNA lessened cholesterol accumulation in NPC1*^mut^* cells (Figure 6F). The introduction of NPC1 a.799/800 C > A and NPC1 a.799/800/816 C > A mutations increased cholesterol accumulation in both NPC1*^wt^* cells (Figures 6C,D) and NPC1*^mut^* cells (Figures 6G,H).

**FIGURE 7:**
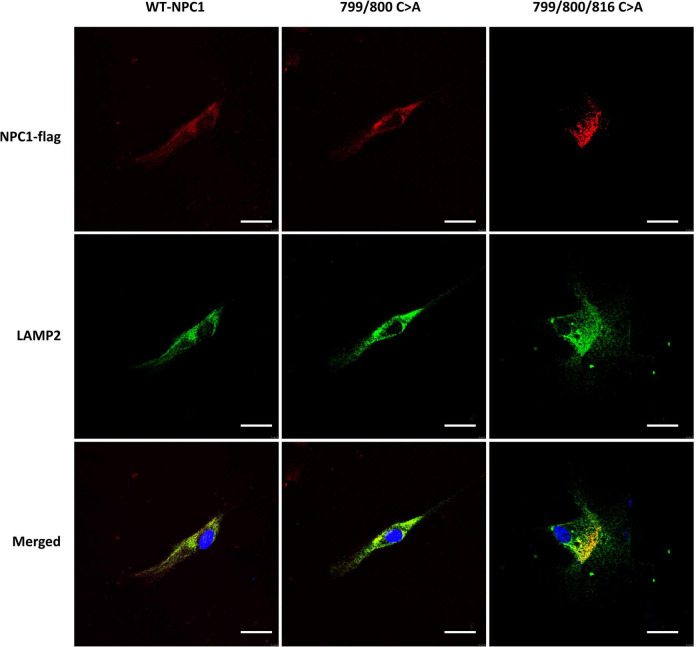
Intracellular localization of mutated NPC1 protein in NPC1^mut^ fibroblasts. **Left column**: NPC1^mut^ fibroblasts transfected with wildtype NPC1 protein (WT NPC1). Middle column: NPC1^mut^ fibroblasts transfected with a.799/800 C > A mutation. **Right column**: NPC1^mut^ fibroblasts transfected with a.799/800/816 C > A mutation. The transfected NPC1 proteins were tagged with red fluorescence **(top panels)** and stained with LAMP2 (green fluorescence, **middle panels**). The pictures were merged to show the co-localization of the proteins **(bottom panel)**.

To further confirm the lysosomal accumulation of cholesterol in the above cells, we performed fluorescent staining analysis in NPC1*^mut^* cells transfected with double and triple mutation. We found that both mutated proteins co-localized with lysosome-associated membrane protein 2 (LAMP2), a known lysosome marker (Figure 7). Collectively, the results showed that Cys at amino acids 799, and 800 were required for the cholesterol transport activity of NPC1, however, they are not required for membrane localization of the protein.

### Palmitoylation defect in Niemann-Pick type C1 is associated with increased intracellular cholesterol accumulation in ARPE19 cells

**FIGURE 8:**
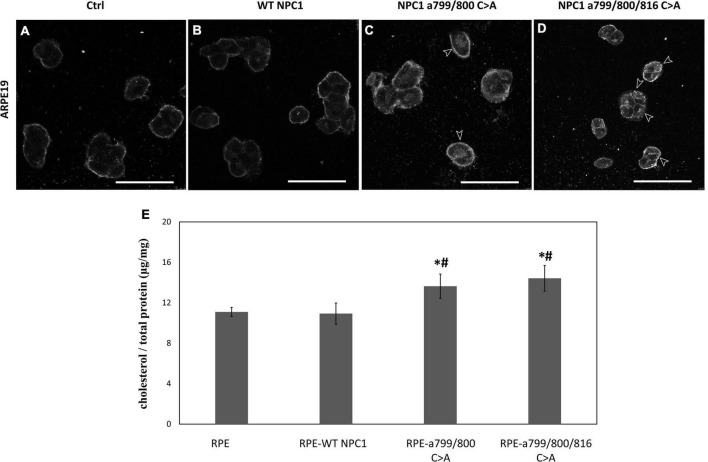
Cholesterol accumulation in ARPE19 cells expressing wildtype and mutated NPC1 proteins. **(A–D)** Filipin staining of ARPE19 cells transfected with wildtype (WT NPC1) and mutated NPC1 as indicated. IN mock- and wildtype NPC1-transfected cells, only plasma membrane showed discernable cholesterol labeling. The introduction of double- and triple-mutated NPC1 caused visible intracellular cholesterol accumulation as indicated by arrows. **(E)** Quantification of intracellular cholesterol in normal ARPE19 cells (RPE) and after wildtype (RPE-WT NPC1), and mutated NPC1 transfection as indicated. The scale bar denotes 50 μm. *Indicates a significant difference when compared to normal PRE cells and ^#^Indicates a significant difference when compared to RPE-WT NPC1 cells (*p* < 0.05 by unpaired *t*-test).

To further validate that Cys 799 and 800 of NPC1 are required for cholesterol transport in RPE cells, the wildtype NPC1 cDNA, NPC1 a.799/800 C > A, and NPC1 a.799/800/816 C > A cDNA were transfected into ARPE19 cell and cholesterol accumulation was analyzed (Figure 8). In ARPE19 cells with mock-transfection and wildtype NPC1 transfection, only the plasma membrane showed faint Filipin staining. However, when the double- and triple-mutated protein was introduced, significant accumulation of cholesterol was observed in RPE cells, indicating impaired lysosomal cholesterol transport. Consistently, cholesterol quantification showed increased concentrations in cells transfected with double and triple mutated NPC1.

## Discussion

Retinal pigment epithelial is an integral part of the retina, both structurally and functionally. RPE cells are involved in the visual cycle, light absorption, nutrient transportation, and photoreceptor cell outer segment phagocytosis (Sparrow et al., 2010; Ao et al., 2017). Dysfunctional RPE is the major cause of age-related retinal degeneration observed in the elderly population (Fleckenstein et al., 2021; Lewandowski et al., 2021; Yang et al., 2021). In this study, we identified 192 potentially palmitoylated proteins. These proteins are particularly enriched in processes and pathways that are related to cell-cell recognition, cell-cell and cell-matrix adhesion, cellular localization, and translation. While many of the proteins identified in this study and the processes they are involved are shared by other cells, we found several palmitoylation-enriched pathways that are of special significance to RPE cell function, such as CXCR4 signaling pathway and Rap1 signaling pathway. We also identified several potentially palmitoylated proteins that have not been reported previously, therefore expanding the repertoire of targets that could be palmitoylated. Some of these proteins, such as Slit2 and OXTR, have special functions in the retina and RPE cells (Ringstedt et al., 2000; Halbach et al., 2015; York et al., 2017; Zhou et al., 2017; Tsuji et al., 2020). Our findings helped in understanding the regulatory mechanism of these proteins in RPE cells. Collectively, our study showed that palmitoylation is an important protein modification in RPE cells.

There have been significant advancements in methodology for large-scale identification of palmitoylated proteins in recent years (Gao and Hannoush, 2017). One of the successful methods is the acyl-biotin exchange (ABE) approach as we used here (Edmonds et al., 2017). After TCEP treatment and NEM protection of freed–SH groups, hydroxylamine is commonly used to cleave thioester bonds. This is followed by the capture of the free thiol by biotinylated thiol reagent and purified by affinity binding to immobilized streptavidin. The enriched proteins are then digested and prepared for LC-MS/MS based protein identification. Another method involves the use of alkynylated fatty acid as a palmitate analogue (Gao and Hannoush, 2017). The alkyne moiety is used to react with an azide-reporter tag using Huisgen’s cycloaddition reaction (Click chemistry). Such labeled proteins could be visualized *in situ* or on SDS-PAGE, or identified using MS-based methods. Each method has its pros and cons. The metabolic labeling method may not catch proteins in low abundance and those with stable palmitoylation and long half-life. The ABE method may result in false-positive candidate proteins since it requires the complete blockage of all reduced cysteines to eliminate false-positives, as well as highly efficient thioester hydrolysis and disulfide-exchange reactions to label and identify palmitoylated proteins (Martin and Cravatt, 2009). Furthermore, S-acylated proteins can be modified by fatty acids other than palmitate (Hallak et al., 1994; Liang et al., 2001). To minimize falsely identified palmitoyl protein candidates, we used the control group at HA hydrolysis step and strict cut-off values (FDR < 1%, peptide confidence level >95%) in this study (Yang et al., 2010). However, to finally prove the existence of palmitoylation modification of a particular protein, biological verification is necessary.

In this study, we confirmed that Cys 799 and 800 of NPC1 were important for its cholesterol transport activity. NPC1 mutation is responsible for up to 95% of the Niemann-Pick type C disease (OMIM 257220), and it is also a cellular gateway protein for the Ebola virus (Gong et al., 2016). NPC1 deficient mouse showed signs of retinal degeneration including lipofuscin accumulation in the pigment epithelium and impaired electroretinography (Claudepierre et al., 2010). Due to its biological importance, significant effort was devoted to understanding the mechanism by which it transports cholesterol. It was found that NPC1 protein has an internal 2-fold pseudosymmetrical configuration (Gong et al., 2016; Li et al., 2016a,b). The transmembrane domains (TMD) 3–7 form a cross membrane channel with an opening large enough for cholesterol molecules to pass, and the TMD 8–13 also form a similar structure. These two clusters of TMD were linked by a stretch of 52 amino acids between TMD 7 and 8 (Gong et al., 2016; Pfeffer, 2016). The palmitoylation sites we identified in this study were located in the middle of this link. A recent study identified a relative “mobile” stretch of amino acids at 800–813 in NPC1 and demonstrated that it is needed for its function (Saha et al., 2020). Deletion of amino acids 807–811 rendered the mutated protein incapable of rescuing cholesterol accumulation in NPC1^–/–^ HeLa cells, while the intracellular localization of the protein remains correct. Our results are in agreement with their observations and provided further information on the configuration of NPC1 in the link region. We hypothesize that the palmitoylation at Cys 799, 800, and 816 anchors the link region to the membrane, stabilizing the transmembrane domains while in the meantime, allowing certain flexibility to the stretch of amino acids in-between. By disrupting the palmitoylation at these sites, the NPC1 protein may lose the configuration needed for sterol sensing and transport. However, it is clear that this stretch of amino acids is not needed for the correct membrane localization of the protein. To further collaborate with our results, mutation of Cys800 to Arg was found in patients with ataxia and atrophy in multiple regions of the brain (Anheim et al., 2014).

In summary, this study expanded the list of palmitoylated proteins to RPE cells and showed that it is an important regulatory mechanism for many RPE cell functions. Although only detergent-resistant lipid raft was analyzed here, our results revealed the enrichment of palmitoylated proteins in processes that are of particular importance to RPE cells. Our results thus target for further study to determine the effect of palmitoylation on specific proteins in RPE cells.

## Data availability statement

The datasets presented in this study are deposited in the ProteomeXchange with identifier PXD034896.

## Author contributions

JKL and YR conducted the RPE cell experiment. SK performed the proteomic analysis. JL and LZ conceived the project, performed the data analysis, wrote the manuscript, and procured the funding’s. All authors contributed to the article and approved the submitted version.

## Footnotes

1
www.swisspalm.org/protiens/O15118

2
https://metascape.org

3
www.swisspalm.org

## References

[B1] Anheim M. Lagha-Boukbiza O. Fleury-Lesaunier M. C. Valenti-Hirsch M. P. Hirsch E. Gervais-Bernard H. (2014). Heterogeneity and frequency of movement disorders in juvenile and adult-onset niemann-pick C disease. *J. Neurol.* 261 174–179. 10.1007/s00415-013-7159-9 24178705

[B2] Ao J. Wood J. P. Chidlow G. Gillies M. C. Casson R. J. (2017). Retinal pigment epithelium in the pathogenesis of age-related macular degeneration and photobiomodulation as a potential therapy? *Clin. Exp. Ophthalmol.* 46 670–686. 10.1111/ceo.13121 29205705

[B3] Bhutto I. A. McLeod D. S. Merges C. Hasegawa T. Lutty G. A. (2006). Localisation of Sdf-1 and its receptor Cxcr4 in retina and choroid of aged human eyes and in eyes with age related macular degeneration. *Br. J. Ophthalmol.* 90 906–910. 10.1136/bjo.2006.090357 16597663 PMC1857162

[B4] Claudepierre T. Paques M. Simonutti M. Buard I. Sahel J. Maue R. A. (2010). Lack of niemann-pick type c1 induces age-related degeneration in the mouse retina. *Mol. Cell Neurosci.* 43 164–176. 10.1016/j.mcn.2009.10.007 19883762

[B5] Crane I. J. Wallace C. A. McKillop-Smith S. Forrester J. V. (2000). Cxcr4 receptor expression on human retinal pigment epithelial cells from the blood-retina barrier leads to chemokine secretion and migration in response to stromal cell-derived factor 1α. *J. Immunol.* 165 4372–4378. 10.4049/jimmunol.165.8.4372 11035074

[B6] Davda D. El Azzouny M. A. Tom C. T. Hernandez J. L. Majmudar J. D. Kennedy R. T. (2013). Profiling targets of the irreversible palmitoylation inhibitor 2-bromopalmitate. *ACS Chem. Biol.* 8 1912–1917. 10.1021/cb400380s 23844586 PMC3892994

[B7] Edmonds M. J. Geary B. Doherty M. K. Morgan A. (2017). Analysis of the brain palmitoyl-proteome using both acyl-biotin exchange and acyl-resin-assisted capture methods. *Sci. Rep.* 7:3299. 10.1038/s41598-017-03562-7 28607426 PMC5468251

[B8] Fleckenstein M. Keenan T. D. L. Guymer R. H. Chakravarthy U. Schmitz-Valckenberg S. Klaver C. C. (2021). Age-related macular degeneration. *Nat. Rev. Dis. Primers* 7:31. 10.1038/s41572-021-00265-2 33958600 PMC12878645

[B9] Fukata Y. Fukata M. (2010). Protein palmitoylation in neuronal development and synaptic plasticity. *Nat. Rev. Neurosci.* 11 161–175. 10.1038/nrn2788 20168314

[B10] Gao X. Hannoush R. N. A. (2017). Decade of click chemistry in protein palmitoylation: Impact on discovery and new biology. *Cell Chem. Biol.* 25 236–246. 10.1016/j.chembiol.2017.12.002 29290622

[B11] Gong X. Qian H. Zhou X. Wu J. Wan T. Cao P. (2016). Structural insights into the niemann-pick C1 (Npc1)-mediated cholesterol transfer and ebola infection. *Cell* 165 1467–1478. 10.1016/j.cell.2016.05.022 27238017 PMC7111323

[B12] Halbach P. Pillers D. A. York N. Asuma M. P. Chiu M. A. Luo W. (2015). Oxytocin expression and function in the posterior retina: A novel signaling pathway. *Invest. Ophthalmol. Vis. Sci.* 56 751–760. 10.1167/iovs.14-15646 25593022 PMC4554231

[B13] Hallak H. Muszbek L. Laposata M. Belmonte E. Brass L. F. Manning D. R. (1994). Covalent binding of arachidonate to G protein alpha subunits of human platelets. *J. Biol. Chem.* 269 4713–4716. 8106438

[B14] Jacobs N. L. Andemariam B. Underwood K. W. Panchalingam K. Sternberg D. Kielian M. (1997). Analysis of a chinese hamster ovary cell mutant with defective mobilization of cholesterol from the plasma membrane to the endoplasmic reticulum. *J. Lipid Res.* 38 1973–1987. 9374120

[B15] Jansen M. Beaumelle B. (2022). How palmitoylation affects trafficking and signaling of membrane receptors. *Biol. Cell* 114 61–72. 10.1111/boc.202100052 34738237

[B16] Ji B. Skup M. (2021). Roles of palmitoylation in structural long-term synaptic plasticity. *Mol. Brain* 14:8. 10.1186/s13041-020-00717-y 33430908 PMC7802216

[B17] Kwon W. Freeman S. A. (2020). Phagocytosis by the retinal pigment epithelium: Recognition. Resolution, recycling. *Front. Immunol* 11:604205. 10.3389/fimmu.2020.604205 33281830 PMC7691529

[B18] Lewandowski D. Sander C. L. Tworak A. Gao F. Xu Q. Skowronska-Krawczyk D. (2021). Dynamic lipid turnover in photoreceptors and retinal pigment epithelium throughout life. *Prog. Retin. Eye Res.* 89:101037. 10.1016/j.preteyeres.2021.101037 34971765 PMC10361839

[B19] Li J. Zhang R. Wang C. Wang X. Xu M. Ma J. (2018). Activation of the small Gtpase rap1 inhibits choroidal neovascularization by regulating cell junctions and ros generation in rats. *Curr. Eye Res.* 43 934–940. 10.1080/02713683.2018.1454477 29601231

[B20] Li X. Saha P. Li J. Blobel G. Pfeffer S. R. (2016a). Clues to the mechanism of cholesterol transfer from the structure of Npc1 middle lumenal domain bound to Npc2. *Proc. Natl. Acad. Sci. U.S.A.* 113 10079–10084. 10.1073/pnas.1611956113 27551080 PMC5018801

[B21] Li X. Wang J. Coutavas E. Shi H. Hao Q. Blobel G. (2016b). Structure of human niemann-pick C1 protein. *Proc. Natl. Acad. Sci. U.S.A.* 113 8212–8217. 10.1073/pnas.1607795113 27307437 PMC4961162

[B22] Liang X. Nazarian A. Erdjument-Bromage H. Bornmann W. Tempst P. Resh M. D. (2001). Heterogeneous fatty acylation of Src family kinases with polyunsaturated fatty acids regulates raft localization and signal transduction. *J. Biol. Chem.* 276 30987–30994. 10.1074/jbc.M104018200 11423543

[B23] Linder M. E. Deschenes R. J. (2007). Palmitoylation: Policing protein stability and traffic. *Nat. Rev. Mol. Cell Biol.* 8 74–84. 10.1038/nrm2084 17183362

[B24] Martin B. R. Cravatt B. F. (2009). Large-scale profiling of protein palmitoylation in mammalian cells. *Nat. Methods* 6 135–138. 10.1038/nmeth.1293 19137006 PMC2775068

[B25] Maziere J. C. Maziere C. Mora L. Routier J. D. Polonovski J. (1982). In situ degradation of sphingomyelin by cultured normal fibroblasts and fibroblasts from patients with niemann-pick disease type a and C. *Biochem. Biophys. Res. Commun.* 108 1101–1106. 10.1016/0006-291x(82)92113-1 7181884

[B26] Morrison E. Kuropka B. Kliche S. Brugger B. Krause E. Freund C. (2015). Quantitative analysis of the human T cell palmitome. *Sci. Rep.* 5:11598. 10.1038/srep11598 26111759 PMC4650600

[B27] Ning W. Jiang P. Guo Y. Wang C. Tan X. Zhang W. (2021). Gps-Palm: A deep learning-based graphic presentation system for the prediction of S-Palmitoylation sites in proteins. *Brief. Bioinform.* 22 1836–1847. 10.1093/bib/bbaa038 32248222

[B28] Percival B. C. Gibson M. Wilson P. B. Platt F. M. Grootveld M. (2020). Metabolomic studies of lipid storage disorders, with special reference to niemann-pick type C disease: A critical review with future perspectives. *Int. J. Mol. Sci.* 21:2533. 10.3390/ijms21072533 32260582 PMC7178094

[B29] Pfeffer S. R. (2016). Clues to Npc1-mediated cholesterol export from lysosomes. *Proc. Natl. Acad. Sci. U.S.A.* 113 7941–7943. 10.1073/pnas.1608530113 27410046 PMC4961152

[B30] Pfeffer S. R. (2019). Npc intracellular cholesterol transporter 1 (Npc1)-mediated cholesterol export from lysosomes. *J. Biol. Chem.* 294 1706–1709. 10.1074/jbc.TM118.004165 30710017 PMC6364775

[B31] Pikuleva I. A. Curcio C. A. (2014). Cholesterol in the retina: The best is yet to come. *Prog. Retin. Eye Res.* 41 64–89. 10.1016/j.preteyeres.2014.03.002 24704580 PMC4058366

[B32] Qu M. Zhou X. Wang X. Li H. (2021). Lipid-Induced S-Palmitoylation as a vital regulator of cell signaling and disease development. *Int. J. Biol. Sci.* 17 4223–4237. 10.7150/ijbs.64046 34803494 PMC8579454

[B33] Ramachandra Rao S. Fliesler S. J. (2021). Cholesterol homeostasis in the vertebrate retina: Biology and pathobiology. *J. Lipid Res.* 62:100057. 10.1194/jlr.TR120000979 33662384 PMC8010701

[B34] Ringstedt T. Braisted J. E. Brose K. Kidd T. Goodman C. Tessier-Lavigne M. (2000). Slit Inhibition of retinal axon growth and its role in retinal axon pathfinding and innervation patterns in the diencephalon. *J. Neurosci.* 20 4983–4991. 10.1523/JNEUROSCI.20-13-04983.2000 10864956 PMC6772277

[B35] Rosenbaum A. I. Maxfield F. R. (2011). Niemann-Pick type C disease: Molecular mechanisms and potential therapeutic approaches. *J. Neurochem.* 116 789–795. 10.1111/j.1471-4159.2010.06976.x 20807315 PMC3008286

[B36] Saha P. Shumate J. L. Caldwell J. G. Elghobashi-Meinhardt N. Lu A. Zhang L. (2020). Inter-domain dynamics drive cholesterol transport by Npc1 and Npc1l1 proteins. *Elife* 9:e57089. 10.7554/eLife.57089 32410728 PMC7228765

[B37] Salaun C. Greaves J. Chamberlain L. H. (2010). The intracellular dynamic of protein palmitoylation. *J. Cell Biol.* 191 1229–1238. 10.1083/jcb.201008160 21187327 PMC3010063

[B38] Sanders S. S. Martin D. D. Butland S. L. Lavallee-Adam M. Calzolari D. Kay C. (2015). Curation of the mammalian palmitoylome indicates a pivotal role for palmitoylation in diseases and disorders of the nervous system and cancers. *PLoS Comput. Biol.* 11:e1004405. 10.1371/journal.pcbi.1004405 26275289 PMC4537140

[B39] Serwa R. A. Abaitua F. Krause E. Tate E. W. O’Hare P. (2015). Systems analysis of protein fatty acylation in herpes simplex virus-infected cells using chemical proteomics. *Chem. Biol.* 22 1008–1017. 10.1016/j.chembiol.2015.06.024 26256475 PMC4543063

[B40] Sparrow J. R. Hicks D. Hamel C. P. (2010). The retinal pigment epithelium in health and disease. *Curr. Mol. Med.* 10 802–823. 21091424 10.2174/156652410793937813 PMC4120883

[B41] Tsuji T. Inatani M. Tsuji C. Cheranov S. M. Kadonosono K. (2020). Oxytocin induced epithelium-mesenchimal transition through rho-rock pathway in Arpe-19 cells, a human retinal pigmental cell line. *Tissue Cell* 64:101328. 10.1016/j.tice.2019.101328 32473703

[B42] Vance J. E. (2006). Lipid imbalance in the neurological disorder. Niemann-Pick C disease. *FEBS Lett.* 580 5518–5524. 10.1016/j.febslet.2006.06.008 16797010

[B43] Veleri S. Lazar C. H. Chang B. Sieving P. A. Banin E. Swaroop A. (2015). Biology and therapy of inherited retinal degenerative disease: Insights from mouse models. *Dis. Model Mech.* 8 109–129. 10.1242/dmm.017913 25650393 PMC4314777

[B44] Wan J. Roth A. F. Bailey A. O. Davis N. G. (2007). Palmitoylated proteins: Purification and identification. *Nat. Protoc.* 2 1573–1584. 10.1038/nprot.2007.225 17585299

[B45] Wang H. Han X. Bretz C. A. Becker S. Gambhir D. Smith G. W. (2016). Retinal pigment epithelial cell expression of active rap 1a by scaav2 inhibits choroidal neovascularization. *Mol. Ther. Methods Clin. Dev.* 3:16056. 10.1038/mtm.2016.56 27606349 PMC4996131

[B46] Wang H. Jiang Y. Shi D. Quilliam L. A. Chrzanowska-Wodnicka M. Wittchen E. S. (2014). Activation of rap1 inhibits Nadph oxidase-dependent ros generation in retinal pigment epithelium and reduces choroidal neovascularization. *FASEB J.* 28 265–274. 10.1096/fj.13-240028 24043260 PMC3868836

[B47] Wilson J. P. Raghavan A. S. Yang Y. Y. Charron G. Hang H. C. (2011). Proteomic analysis of fatty-acylated proteins in mammalian cells with chemical reporters reveals S-Acylation of histone H3 variants. *Mol. Cell Proteomics* 10:M110001198. 10.1074/mcp.M110.001198 21076176 PMC3047146

[B48] Wittchen E. S. Hartnett M. E. (2011). The small gtpase rap1 is a novel regulator of rpe cell barrier function. *Invest. Ophthalmol. Vis. Sci.* 52 7455–7463. 10.1167/iovs.11-7295 21873678 PMC3183976

[B49] Yan X. Ma L. Hovakimyan M. Lukas J. Wree A. Frank M. (2014). Defects in the retina of niemann-pick type C 1 mutant mice. *BMC Neurosci.* 15:126. 10.1186/s12868-014-0126-2 25472750 PMC4267119

[B50] Yañez M. J. Marín T. Balboa E. Klein A. D. Alvarez A. R. Zanlungo S. (2020). Finding pathogenic commonalities between niemann-pick type c and other lysosomal storage disorders: Opportunities for shared therapeutic interventions. *Biochim. Biophys. Acta Mol. Basis Dis.* 1866:165875. 10.1016/j.bbadis.2020.165875 32522631

[B51] Yang S. Zhou J. Li D. (2021). Functions and diseases of the retinal pigment epithelium. *Front. Pharmacol.* 12:727870. 10.3389/fphar.2021.727870 34393803 PMC8355697

[B52] Yang W. Di Vizio D. Kirchner M. Steen H. Freeman M. R. (2010). Proteome scale characterization of human S-Acylated proteins in lipid raft-enriched and non-raft membranes. *Mol. Cell Proteomics* 9 54–70. 10.1074/mcp.M800448-MCP200 19801377 PMC2808267

[B53] York N. Halbach P. Chiu M. A. Bird I. M. Pillers D. M. Pattnaik B. R. (2017). Oxytocin (Oxt)-Stimulated inhibition of Kir7.1 activity is through pip(2)-Dependent Ca(2+) response of the oxytocin receptor in the retinal pigment epithelium in vitro. *Cell Signal.* 37 93–102. 10.1016/j.cellsig.2017.06.005 28603013 PMC5554455

[B54] Zhou W. Wang H. Yu W. Xie W. Zhao M. Huang L. (2017). The expression of the slit-robo signal in the retina of diabetic rats and the vitreous or fibrovascular retinal membranes of patients with proliferative diabetic retinopathy. *PLoS One* 12:e0185795. 10.1371/journal.pone.0185795 28973045 PMC5626485

[B55] Zhou Y. Zhou B. Pache L. Chang M. Khodabakhshi A. H. Tanaseichuk O. (2019). Metascape provides a biologist-oriented resource for the analysis of systems-level datasets. *Nat. Commun.* 10:1523. 10.1038/s41467-019-09234-6 30944313 PMC6447622

